# HIDING IN PLAIN SITE: LOW BACK PAIN RATES IN OLDER ADULTS WITH LUMBAR SPINAL STENOSIS

**DOI:** 10.1093/geroni/igad104.2912

**Published:** 2023-12-21

**Authors:** Beth Hogans, Bernadette Siaton, Stuart Goldman, John Sorkin

**Affiliations:** VAMHCS / Johns Hopkins School of Medicine, Baltimore, Maryland, United States; University of MD School of Medicine, Baltimore, Maryland, United States; Sinai/Life Bridge, Pikesville, Maryland, United States; VAMHCS/UMB, Baltimore, Maryland, United States

## Abstract

**Introduction:**

Lumbar spinal stenosis (LSS) is highly prevalent in older adults, negatively impacting mobility and quality of life. Typically associated with progressive impairment of walking distance and pain which radiates into the legs, there is persistent controversy regarding the extent to which low back pain (LBP) is associated with LSS.

**Objectives:**

To evaluate population-level evidence for and strength of an association of LBP with LSS in older adults. Data Source: Medicare Part B Carrier and Outpatient claims data, 5% standard analytical file for 2017.

**Methods:**

Unadjusted and adjusted analysis of cross-sectional data, combined with condition process analysis

**Results:**

Several hundred thousand older adults met criteria for inclusion in this study. Lumbar spinal stenosis was common in both older adult males and females, but slightly more commonly diagnosed in females. Low back pain was prevalent in older adults but more so in females. A majority of older adults with LSS were diagnosed with LBP. Unadjusted and adjusted analyses demonstrated that LBP was present at increased rates in older adults with LSS.

**Conclusions:**

Low back pain is diagnosed in the majority of older adults diagnosed with lumbar spinal stenosis. It is important to recognize that the symptom profile of older adults with spinal stenosis includes low back pain and treatment should be adjusted to respond to this management need.

